# Consequences of Misdiagnosed and Mismanaged Hereditary Angioedema Laryngeal Attacks: An Overview of Cases from the Romanian Registry

**DOI:** 10.1155/2018/6363787

**Published:** 2018-10-22

**Authors:** Dumitru Moldovan, Noémi Bara, Valentin Nădășan, Gabriella Gábos, Enikő Mihály

**Affiliations:** ^1^Romanian Network for Hereditary Angioedema, 11a Sântana St, 540256 Tîrgu-Mureş, Romania; ^2^University of Medicine and Pharmacy, Mureş County Hospital, 1 Marinescu St, 540139 Tîrgu-Mureş, Romania; ^3^Mureş County Hospital, 1 Marinescu St, 540103 Tîrgu-Mureş, Romania; ^4^MediQuest Medical Center, Sângeorgiu de Mureș, Romania

## Abstract

Emergency department (ED) physicians frequently encounter patients presenting with angioedema. Most of these involve histamine-mediated angioedema; however, less common forms of angioedema (bradykinin-mediated) also occur. It is vital physicians correctly recognize and treat this; particularly since bradykinin-mediated angioedema does not respond to antihistamines, corticosteroids or epinephrine and hereditary angioedema (HAE) laryngeal attacks can be fatal. Here we present four case reports illustrating how failures in recognizing, managing, and treating laryngeal edema due to HAE led to asphyxiation and death of the patient. Recognition of the specific type of angioedema is critical for rapid and effective treatment of HAE attacks. Bradykinin-mediated angioedema should be efficiently differentiated from the most common histamine-mediated form. Improved awareness of HAE and the associated risk of life-threatening laryngeal edema among emergency physicians, patients, and relatives and clear ED treatment protocols are warranted. Moreover, appropriate treatments should be readily available to reduce fatalities associated with laryngeal edema.

## 1. Introduction

Hereditary angioedema (HAE) is an autosomal dominant-inherited disease estimated to affect around 1 in 50,000 individuals [1]. HAE is caused by a deficiency (Type I) or dysfunction (Type II) in C1 inhibitor (C1-INH) [2]. In patients with HAE, insufficient levels of functional C1-INH lead to reduced inhibition of the kallikrein-kinin system. During an HAE attack, the system is triggered (by minor trauma, drugs, stress, or physical activities) leading to increased levels of bradykinin, which increases vascular permeability and causes angioedema, presenting as skin swellings, abdominal pain, tongue swellings, and upper respiratory airways obstruction (i.e., laryngeal edema) [2].

Laryngeal edema can be potentially life-threatening; affecting up to 48% of patients with HAE, it can lead to asphyxiation and death [3–6]. Evidence from clinical studies and case reports demonstrates that the course of laryngeal edema can be unpredictable. Although the mean interval between initial onset of symptoms and asphyxiation is 7–8 hours [3, 5], cases with extremely short course (i.e., 20 minutes) have been reported [4, 5]. Incidences of fatal laryngeal attacks were first reported by Osler in 1888 [7]. Since then numerous studies and case reports have demonstrated fatalities as a result of laryngeal edema in both diagnosed [4–6, 8, 9] and undiagnosed patients [4–6, 8, 10, 11]. Laryngeal edema has been associated with a mortality rate of 12–40% [4, 5, 9, 12], and in many undiagnosed cases, family history and recurrent skin and abdominal attacks since childhood could have informed the correct diagnosis [5, 6, 8].

Fatal attacks are commonly attributed to delays in receiving treatment and failure in administering effective therapy, often as a result of misdiagnosis. In a case series by Bork et al. [5], fatal laryngeal edema occurred in three patients previously undiagnosed with HAE. In two of the undiagnosed cases, laryngeal edema was misdiagnosed as an allergic reaction, and antihistamine and prednisolone were administered to no avail. Similarly, in a case series described by Bork and Barnstedt [13], all patients failed to receive adequate treatment; in three cases, this was due to a lack of knowledge so patients did not seek help in time. In the fourth case, it was a result of misdiagnosis; the edema was considered allergic by the emergency physicians and intravenous steroids and antihistamines were administered. Furthermore, in studies by Bork et al. [4] and Xu et al. [9], deaths were caused by delays in receiving effective emergency treatment and/or not receiving effective drug therapy, i.e., C1-INH concentrate, ecallantide, or icatibant.

Improved diagnosis and awareness have been shown to reduce mortality rate in patients with HAE [10, 11]. Frank et al. [10] noted that patient and physician education was crucial in improving the outcomes in HAE patients, and Ohela [11] advocated the use of treatment cards with diagnosis and treatment instructions which patients should carry at all times. Treatments for other types of angioedema (i.e., histamine-mediated), such as corticosteroids, antihistamines, and epinephrine, are not effective in treating HAE attacks; thus accurate diagnosis of HAE is particularly important [12, 14, 15]. In particular, C1-INH concentrate has been shown to significantly reduce the duration of laryngeal edemas (treated: 15.3 hours versus untreated: 100.8 hours) [5].

Due to the nature and high risk of mortality associated with laryngeal attacks, the majority of patients seek emergency medical treatment. Most emergency department (ED) physicians are familiar with histamine-mediated angioedema, such as allergic angioedema; however, fewer ED physicians are familiar with the less common bradykinin-mediated angioedema. The cases reviewed above highlight the importance of early and rapid recognition and management of life-threatening laryngeal edema in HAE patients. It is vital therefore that attacks are accurately assessed in the ED in order for patients to receive prompt and effective emergency treatment as soon as possible. Despite efforts to improve the diagnosis and management of HAE in the ED, fatal laryngeal attacks still occur and there is a need to raise awareness and implement existing guidelines for the recognition and management of specific forms of angioedema in the ED [14, 16, 17]. In light of this, we reviewed the cases of 96 patients with C1-INH deficiency enrolled in the Romanian HAE Registry from 2006 onwards. We present a case series of four patients who died from asphyxiation caused by laryngeal edema following an HAE attack. In three cases, the patient had been previously diagnosed with HAE; the fourth case was an undiagnosed patient with a history of recurrent attacks. Each of these cases provides an example of how misdiagnosis, mismanagement, and/or limited access to effective HAE therapy can result in fatalities from laryngeal attacks. These cases provide real-world evidence of the need for greater awareness and management of HAE, particularly in the ED.

## 2. Case Presentation

### 2.1. Case Report 1

Case 1 is for a female patient aged 20 at symptom onset. Despite a positive family history of angioedema attacks, a long history of recurrent peripheral and abdominal attacks, and more than 100 laryngeal attacks, the patient was only diagnosed with HAE at age 50 (Table 1). Typical symptoms included mild peripheral edema of the limbs typically lasting 3 days. Over time skin swelling extended to the abdominal and thoracic walls and head. Some of face edema was followed by aphonia and suffocation. Hours before the onset of an HAE attack, she was always progressively adynamic and these symptoms resolved gradually when the edema became evident. Initially, painful abdominal attacks were rare; however, the patient began to experience weekly abdominal attacks preceded or followed by peripheral edema. The patient repeatedly received hydrocortisone, antihistamines, and epinephrine for the treatment of attacks, all of which were ineffective. Specific treatment with C1-INH concentrate, icatibant, or fresh frozen plasma (FFP) was never received in the ED. Following some of these attacks, the gallbladder, appendix, and left ovarium were surgically removed.

The fatal attack, occurring at age 52, started with dysphagia, quickly followed by dysphonia and dyspnea. The patient was admitted to a small regional hospital and 6 hours after symptom onset underwent respiratory arrest whilst waiting to receive FFP. Resuscitation attempts were unsuccessful and the autopsy revealed laryngeal edema.

Evaluation of the case reveals several failings in the patient's treatment: firstly the severity and type of angioedema attack were not correctly recognized or assessed by the emergency physician. Secondly, the ED did not contact the HAE reference center upon admission of the patient to gather details of any previous history of attacks. Finally, the airway was not secured and emergency measures, such as cricothyrotomy, were not prepared in advance nor performed. As a consequence, life-saving measures and effective drug therapy were not provided early enough to prevent the patient's death.

### 2.2. Case Report 2

A male patient aged 3 at symptom onset was diagnosed with HAE at age 7 (Table 1). Initial symptoms included facial swelling and painful abdominal attacks, occasionally associated with vomiting and/or diarrhea. Most of these attacks were followed in 2–3 days by swelling of a hand, leg, or genitalia. The patient had a positive family history of HAE; his brother, father, grandfather, and one paternal aunt had a history of attacks of recurrent peripheral edema and his grandfather suffered a fatal laryngeal attack aged 67 years. Prior to diagnosis, the patient had an appendectomy at age 6 following an abdominal attack. After diagnosis, the patient received prophylactic treatment with tranexamic acid.

The fatal attack, occurring at age 11, started with facial edema, followed by progressive dysphagia, dysphonia, and dyspnea. He was admitted to a small local hospital and treated for allergic laryngeal edema with repeated doses of corticosteroids and epinephrine, despite his mother advising the treating physician that these treatments had previously been ineffective. Respiratory arrest occurred 3 hours after admission and neither tracheotomy nor intubation was attempted. Autopsy confirmed obstructive laryngeal edema.

Evaluation of the case reveals several failings in the patient's treatment; although the emergency physician was made aware of the nature of the edema, hereditary angioedema was not recognized by the treating physician. The patient's family history and previous history of attacks were also not considered. Consequently, the patient was misdiagnosed and incorrectly treated for allergic edema. Effective treatment was not given and life-saving measures were neither prepared nor attempted.

### 2.3. Case Report 3

A male patient aged 8 at symptom onset was diagnosed with HAE at age 57 (Table 1). Initial symptoms included abdominal attacks recurring every two weeks and the first laryngeal attack occurred at age 16. In the 8 years prior to his death, the patient experienced one laryngeal attack per year; one led to suffocation with loss of consciousness and four required intubation. The patient had been treated with FFP on several previous occasions with a fair response. In the 6 months prior to his death, he had been symptom-free.

The fatal attack, occurring at age 59, started with dysphonia. Attack severity progressed rapidly; the time from symptom onset to respiratory arrest was 20 minutes. Resuscitation measures were undertaken in the hospital; however, this was only after irreversible hypoxic brain damage had occurred. The patient did not have access to on-demand therapy for treatment of attacks at home.

Evaluation of the case reveals that the course of laryngeal edema can be extremely short. It is therefore imperative that patients have appropriate home treatment available, although this may not always be effective for rapidly progressing attacks. Therefore EDs should be aware of the potential for HAE attacks to progress rapidly so that emergency measures to keep the airway safe and/or cricothyrotomy can be urgently administered.

### 2.4. Case Report 4

Case 4 is for a male patient aged 22 at symptom onset (Table 1). The patient was not previously diagnosed with HAE despite experiencing repeated peripheral and abdominal attacks. The patient also had a positive family history; five family members with confirmed HAE had died from laryngeal edema.

The fatal attack occurred at age 42; the patient awoke with the sensation of a lump in his throat and gradually developed dysphagia. After approximately 8 hours, he became dysphonic but refused to attend the ED and was treated at home with corticosteroids by his wife, a nurse. One hour later, he could not swallow and extreme breathlessness followed so the patient attempted a self-tracheotomy. An ambulance was called, and while waiting for it, the patient lost consciousness. Cardiac massage was performed by the attending ambulance crew and 5 mg of epinephrine was administered. As the patient was not intubated because no physician was in attendance, his wife attempted to perform a tracheotomy which was unsuccessful. Upon arrival at the ED, an electrocardiogram indicated electrical activity but the patient remained in respiratory arrest and later died. HAE diagnosis was confirmed retrospectively when the patient's daughter, with similar peripheral edema and abdominal symptoms, was diagnosed with low C1-INH levels.

Evaluation of the case reveals the challenges of treating an undiagnosed HAE patient. Despite previous repeated swelling attacks and a family history of HAE and fatal laryngeal edema, the lack of sufficient education and awareness by the patient and emergency medicine crew led to the mismanagement of the fatal episode. Notably, there was a long delay in calling emergency services and they were who in turn were not prepared to administer measures to keep the upper airway open, perform an emergency cricothyrotomy, or be able to provide effective treatment for the HAE attack.

## 3. Discussion

These cases demonstrate that fatal laryngeal edema can occur regardless of HAE diagnosis, family history, or previous episodes of laryngeal edema. In three of the four cases, the patient had been previously diagnosed with HAE (cases 1, 2, and 3); in the fourth case, the patient was undiagnosed (case 4). All patients had a positive family history of HAE and a clinical history of typical HAE symptoms, including recurrent abdominal and/or peripheral edema since childhood or early adulthood. In three of four cases, the patients had a history of previous laryngeal edema; patient 1 had experienced more than 100 laryngeal edemas, whilst patients 2 and 3 had a history of a few laryngeal attacks. Patient 4 died from his first laryngeal edema; this highlights that any laryngeal edema has the potential to be fatal and thus all should be treated as an emergency. In all cases, misdiagnosis and/or mismanagement of the attack were key factors that contributed to the fatal outcome. In addition to these cases, by appraising relatives of patients in our database we identified a further 24 individuals who had died by asphyxiation. Notably, none of the 24 individuals had undergone analysis of serum C1-INH activity or levels.

Numerous previous cases have demonstrated the risk associated with laryngeal edema. The constant mortality rate of approximately 30% has been attributed to misdiagnosis and mismanagement of bradykinin-mediated angioedema [4, 6, 12]. This includes misdiagnosis of HAE from allergic edema, delays in receiving emergency treatment, and failure to provide effective drug therapies [4]. Since early case reports published in 1962 [12], the need for awareness and education of life-threatening laryngeal edema has been systematically emphasized [3–6, 8–11, 13–19]. Furthermore, as many fatal cases occur in undiagnosed patients who have a family history of HAE, awareness and education among relatives are critical. This facilitates the detection of the early signs of laryngeal edema and prompts the patient to seek medical help so that emergency measures and effective drug therapies can be administered in the early phase (before dyspnea) when timely treatment is most critical [4].

The cases described above demonstrate that fatal laryngeal edema still occurs despite the advent of effective therapies to treat HAE. Importantly these cases emphasize that lack of awareness, misdiagnosis, and mismanagement continue to be contributing factors to the fatal laryngeal attack. In three of the four cases, a diagnosis of HAE was known, and therefore appropriate recognition and treatment should have been received. However, in all cases, appropriate management and treatment of the attack were not received. In the fourth case, lack of awareness of the nature of an HAE attack by the patient, relatives, and emergency services resulted in mismanagement of the attack and contributed to the death of the patient. It is essential to continue raising awareness of the risks of laryngeal attacks, not only because upper airway obstruction has been estimated to account for 30–50% mortality in undiagnosed patients or in those who are improperly managed [1, 4, 20], but also because recent case reports suggest that laryngeal attacks may be associated with additional cerebral complications, such as blindness and tetraspasticity, resulting from hypoxic brain damage [21].

In many cases, patients with laryngeal edema present to the ED; however, in a recent patient survey 99% of patients agreed that management of HAE in the ED needed improvement [22]. Key aspects for improvement included recognition and diagnosis of HAE, understanding of HAE as a serious disease, and appropriate medication management (i.e., use of HAE-targeted therapy rather than corticosteroids, epinephrine, and antihistamines) [22]. In particular, ED staff should be trained to differentiate between the common forms of angioedema (allergic, histamine-mediated) and the less common bradykinin-mediated form, as well as to recognize and appropriately manage each form of angioedema [14–17]. Moreover, effective drugs for the treatment of bradykinin-mediated angioedema should be easily accessible to the ED so that prompt treatment can be provided [14].

Recently, Bernstein et al. [14] published practical guidance for the management and treatment of HAE patients in the ED. This guidance includes advice on diagnosing and treating angioedema in the ED, which could serve as the basis to develop an ED protocol or management plan easily accessed at any ED. In particular, the guidance provides key distinguishing features of different angioedema forms and provides practical guidance on key questions that may prove life-saving, e.g., “airway secure?”, “known/suspected allergy?”, “known HAE?”, and “personal/family history of angioedema?” [14]. In the current case series, all patients had a family history of HAE and 3 out of 4 had a confirmed diagnosis of HAE. This highlights that asking/listening to family members can provide valuable information on previous attacks and family history, potentially increasing the awareness of HAE and the specific therapies available. Moreover, early securing of the airway is a first vital step, which was missed in all the presented cases. Development of clear ED treatment protocols, for example, triage algorithms or individual ED plans, would be beneficial to ensure that critical actions are undertaken in the ED to secure, stabilize, and treat HAE patients in a timely and appropriate manner [14, 16, 17, 22].

Finally in addition to increased awareness, appropriate treatment of HAE attacks, particularly laryngeal attacks, is essential. The availability of appropriate drug therapies, such as C1-INH concentrate, or recombinant, icatibant or ecallantide, is paramount in reducing mortality and improving the quality of life of HAE patients [3, 4, 23]. Despite progress in the past years in the provision of some EDs with C1-INH concentrate and recombinant C1-INH, these resources remain insufficient. Our report highlights that the time from the beginning of upper respiratory symptoms to asphyxiation may be as short as 20 minutes. This observation has previously been reported [3, 4] and emphasizes the need for patients to maintain their own supply of C1-INH concentrate or recombinant, icatibant or ecallantide, at home in order to prevent fatalities [24]. The recent approval of icatibant home treatment for acute attacks in Romania is a crucial step towards reducing and preventing mortality due to HAE laryngeal attacks.

## 4. Conclusions

Due to the unpredictable and potentially fatal nature of laryngeal HAE attacks, rapid and appropriate emergency care is critical. Emergency physicians should be able to recognize the symptoms of HAE attacks, distinguish HAE from other forms of angioedema, and be aware of effective HAE medication. Despite the publication of numerous case series and the recommendation for increased awareness, the cases presented herein demonstrate that misdiagnosis and mismanagement of laryngeal attacks are a continuing challenge. We welcome the recommendations made in recent guidance documents for the ED and hope that the cases presented herein highlight the importance of their implementation. Furthermore, national education programs are essential to raise awareness amongst patients, relatives, and ED physicians and to improve access to appropriate treatments.

## Figures and Tables

**Table 1:** History and clinical data of 4 patients with hereditary angioedema (HAE) who died from laryngeal edema and asphyxiation.

Pt	Gender	Age (years)			HAE attacks			Family history				Treatment received		
		At onset of symptoms	At HAE diagnosis	At death	Frequency of previous abdominal and/or peripheral attacks	No. of previous laryngeal attacks	Unnecessary abdominal surgeries before diagnosis?	No. of known relatives with HAE	Relatives experiencing laryngeal edema?	No. of deaths in family resulting from suspected laryngeal edema?	Interval between onset of laryngeal edema and asphyxiation	Home	Emergency services / Hospital	Emergency procedures administered
1	Female	20	50	52	Weekly	>100	3	6	Yes	-	6 h	-	-	-

2	Male	3	7	11	Monthly	3	1	4	Yes	2	3 h	-	Corticosteroids and epinephrine	-

3	Male	8	57	59	Yearly	10	-	3	Yes	-	20 min	-	-	-

4	Male	22	Not previously diagnosed	42	Twice/ month	0	-	5	Yes	5	11 h	Corticosteroids	Epinephrine	Attempted (self-) tracheotomy

## Data Availability

The details reported in the current case series are available from the corresponding author upon request.
